# 

**Published:** 1887-03-26

**Authors:** 


### CONTENTS.

**Persistence, not Panic** - - - - 429

**Words of Consolation.**—XXVI. Death - - 430

**Poetry.**—Patience - - - - - - 430

**Nurse Marion's Romance.** By KATE FURLEY - 431

**Nurse Farms, Hospitals, and Private Nursing Institutions.** By a Cottage Hospital Nurse, and the Hon. Secretary of the Workhouse Infirmary Nursing Association - - - - - - - 433

**The Alleged Inhumanity at Guy's.** By G. W. POTTER, M.D. - - - - - - 434

**Books, Good and Otherwise** - - - 434

**Questions and Answers** - - - - 434

**National Pension Fund for Hospital Officials and Nurses** - - - - - - 435

**Notes and News.**—Items.—Jubilee Commemorations.—The Mold Cottage Hospital.—University College Hospital.—Hospital Enlargement.—Vacancies.—The late Dr. R. E. Carrington.—Hospital Anecdotes.—The Brompton Cancer Hospital.—The London Dental Hospital.—Hospital Alms-boxes.—Royal Hospital for Diseases of the Chest.—Donations to Medical Charities.—A Newsvendor's Legacy, etc., etc. - - 436

**Annotations.**—Faith Healing.—Alleged Inhumanity at Guy's - - - - - - - 439

**Difficult Cases** - - - - - - 439

**Nursing in Workhouse Infirmaries.** Discussion on Miss Manson's Paper - - - - 440

**Chats about Medical Museums.** By ONE OF THE CROWD.—No. II - - - - - - 442

**In- and Out-Patients' Hospital Diary** - - 443

**The Children's Corner.**—Puzzles, Answers, etc. - vi

**Amusements with Profit** - - - - vi

